# Aortic stiffness in patients with early sepsis

**DOI:** 10.1186/cc13326

**Published:** 2014-03-17

**Authors:** S Kazune, A Grabovskis, E Strīke, I Vanags

**Affiliations:** 1 Hospital of Traumatology and Orthopaedics, Riga, Latvia; 2 Institute of Atomic Physics and Spectroscopy, University of Latvia, Riga, Latvia; 3 Riga Stradins University, Riga, Latvia

## Introduction

Acute and chronic systemic inflammatory conditions are associated with aortic stiffening. Carotid-femoral pulse wave velocity (PWV), a marker of aortic stiffness, increases in patients with inflammatory diseases and independently correlates to levels of C-reactive protein (CRP). The effects of massive inflammatory response in early sepsis on mechanical properties of the aorta have not been investigated. The objective of the current study was to prospectively assess aortic stiffness in patients with early severe sepsis and septic shock and relate it to inflammatory and haemodynamic variables and outcome.

## Methods

We recruited patients meeting criteria for severe sepsis and septic shock within 24 hours of admission to ICU. After haemodynamic stabilisation, PWV was recorded at inclusion and after 48 hours using dual-channel plethysmography. Severity of illness was assessed with APACHE II and serial SOFA scores, haemodynamic and inflammatory parameters (CRP, procalcitonin and fibrinogen) recorded. A 28-day follow-up was performed to distinguish between survivors and nonsurvivors.

## Results

Twenty consecutive general ICU patients (six with severe sepsis and 14 with septic shock) were enrolled in the study; median age 59 years (IQR 56.5 to 72), APACHE II score 17 (13 to 20.5), SOFA score 5 (IQR 4 to 9). At 28 days, six patients had died. Median initial PWV was 10.4 (IQR 6.9 to 12.1) m/second in patients with severe sepsis, and 6.8 (IQR 5.3 to 7.5) m/second in patients with septic shock (*P *= 0.13). After 48 hours, PWV in the severe sepsis and septic shock groups had become similar, 9.3 (IQR 7.3 to 11.1) m/second and 9.2 (IQR 7.8 to 13) m/second respectively (*P *= 0.96). PWV had significantly increased in survivors (7.8 to 12.3 m/second) (*P *= 0.04) versus nonsurvivors (6 to 7.8 m/second) (*P *= 0.69). Higher PWV correlated with increasing systolic pressure and lower CRP levels (*r *= 0.73, *P *= 0.01).

## Conclusion

In early sepsis, aortic stiffness is decreased in patients with greater disease severity, and in survivors increases to median levels within 48 hours. The main factors associated with lower pulse wave velocity are lower systolic pressure and higher CRP levels. The association of high serum CRP levels with low aortic stiffness in patients with sepsis does not match data described in the literature [1].
